# Extensive Characterization of Alginate, Chitosan and Microfibrillated Cellulose Cast Films to Assess their Suitability as Barrier Coating for Paper and Board

**DOI:** 10.3390/polym15163336

**Published:** 2023-08-08

**Authors:** Anna Mayrhofer, Samir Kopacic, Wolfgang Bauer

**Affiliations:** Institute of Bioproducts and Paper Technology, Graz University of Technology, 8010 Graz, Austria; samir.kopacic@tugraz.at (S.K.); wolfgang.bauer@tugraz.at (W.B.)

**Keywords:** biopolymer, chitosan, alginate, microfibrillated cellulose, solution cast film, barrier, coating

## Abstract

The vast amount of synthetic polymers used in packaging is putting a strain on the environment and is depleting finite, non-renewable raw materials. Abundantly available biopolymers such as alginate, chitosan and microfibrillated cellulose (MFC) have frequently been suggested in the literature to replace synthetic polymers and their barrier properties have been investigated in detail. Many studies aim to improve the properties of standalone biopolymer films. Some studies apply these biopolymers as barrier coatings on paper, but the solids content in most of these studies is quite low, which in turn would result in a high energy demand in industrial drying processes. The aim of this study is to suggest a laboratory procedure to investigate the suitability of these biopolymers at higher and such more industrially relevant solids content as potential coating materials for paper and board in order to improve their barrier properties. First, biopolymer solutions are prepared at a high solids content at which the viscosity at industrially relevant higher shear rates of 50,000 s^−1^ (1000 s^−1^ for MFC) is in the same range as a synthetic reference material (in this case ethylene vinyl alcohol EVOH) at 10 wt%. These solutions are analyzed regarding properties such as rheology and surface tension that are relevant for their coatability in industrial coating processes. Then, free-standing films are cast, and the films are characterized regarding important properties for packaging applications such as different surface, mechanical and barrier properties. Based on these results suitable biopolymers for future coating trials can be easily identified.

## 1. Introduction

With the worldwide growth in population and the concentration in cities, the demand for packaging materials is steadily increasing. This results in multiple challenges concerning the handling of finite fossil-based resources and collection and treatment of the packaging materials after their intended use [1]. To counteract these negative impacts, the Directive 2018/852 of the European Parliament was issued [2]. Encouraged by this directive, the use of biobased and biodegradable materials for packaging applications is gaining more and more attention both in research and industry. Due to this, paper and board has become an even more interesting packaging material because of its origin from renewable resources, recyclability and biodegradability.

Especially in the food industry, packaging additionally plays an important role to reduce food waste and to extend the shelf life of perishable goods. Adequate protection of goods requires mechanical stability of the packaging and suitable barrier properties. Paper and board can provide the required mechanical stability but fall short in providing sufficient barrier properties. Required barrier properties strongly depend on the type of packaged food. The demand for the barrier function of packaging intended for fruits and vegetables is comparatively low, with oxygen transmission rates (OTR) between 10,000 and 100,000 cm³ STP/(m²·d·bar) at standard temperature and pressure (STP) of 23 °C and 0 % RH and water vapor transmission rates (WVTR) between 10 and 1500 g/(m²·d). Other food groups like milk and milk products require OTR between 100 and 10,000 cm^3^ STP/(m^2^*d*bar) and low WVTR of less than 10 g/(m²·d). Oils, vacuum packed coffee, nuts and snacks require equally low WVTR and OTR below 10 cm³ STP/(m²·d·bar) [3]. In 2015, the types of plastic most used in different kinds of packaging were polyethylene (PE), polypropylene (PP) and polyethylene terephthalate (PET). Other kinds of synthetic materials often applied in packaging applications are polyvinyl alcohol (PVOH) and ethylene vinyl alcohol (EVOH), which are usually not used as a sole packaging material but as coating material to increase barrier properties especially against oxygen transmission [3].

Polysaccharides such as alginate, chitosan and microfibrillated cellulose (MFC) appear to be suitable alternatives for these petroleum-based coating materials because of their barrier properties and could therefore help to reduce the amount of non-renewable material used in the packaging industry.

Alginate is an unbranched hydrophilic polysaccharide produced from brown algae, which can be found in large amounts in nature [4]. In general, films formed from alginate have good oxygen barrier properties but form poor water vapor and moisture barriers [5,6,7]. Multiple studies investigate the properties of alginate films and their potential use in the packaging industry mainly focusing on alginate films with additional components like plasticizers [5,8,9], linseed oil [6], aloe vera [10] or composite films [9,11]. All these modifications bring different benefits to the film and barrier properties but do not allow for the direct comparison between materials. Therefore, investigating the property of the material on its own is necessary.

Chitosan is the deacetylated derivate of chitin, which is sourced from crustacean shell waste, and is one of the most abundant polysaccharides in nature second to cellulose [12]. Commonly, films made from chitosan have low water barrier properties due to their high hydrophilicity [13,14,15,16], but like alginate, they are good oxygen barriers [16,17,18]. Most studies investigating film formation use plasticizers to alter film properties [13,19]. Other studies focus on the solution concentration used to cast films and add surfactant [20] or investigate films containing additional starch [14,15] or acetate [21]. With these modifications again the direct assessment of different materials is not possible.

MFC is mainly produced from bleached cellulosic pulp by mechanical treatments like a homogenizer, microfluidizer or via a grinding process [22]. Films produced from this material show good barrier properties against air and oxygen [23]. These properties depend on multiple factors like the source and production of MFC [24,25], and the preparation of films and many studies focus on their improvement [20,21].

The properties of solution cast films strongly depend on the preparation and drying method and the addition of other components. The solids content of the casting solution is kept below 1 wt% in most studies. Some studies use higher concentrations, but this results in a limitation regarding how thin the films can be produced [26,27,28]. In general, it was observed that the water and oxygen permeability decreases with higher drying temperature [8,17,20,29]. The influence of temperature on the mechanical properties depends on the addition of plasticizer, but without its addition, the mechanical properties of alginate [17] and chitosan [19] films decrease with increased drying temperature. Whilst many studies cited above focus on improving certain film and barrier properties for one biopolymer, an overall characterization and comparison of different biopolymers films prepared under the same conditions and referenced to a state-of-the-art packaging material can be used to determine the principal suitability of individual materials for packaging. As a next step, the application of biopolymer coatings on paper or board is of interest. To estimate achievable barrier properties data from these films can be used as guidelines. Often coatings are performed without additional additives [30,31,32,33,34]; therefore, the correlation between solution cast films without and containing specific additives is difficult.

Another important factor in the coating process that has not been researched in sufficient depth are the properties of the above-mentioned biobased coating materials. Because of their high viscosities in aqueous solutions or dispersions [35,36,37,38] materials like alginate, chitosan or MFC are commonly used at very low solid content for coating of paper in research [30,31,32,33,34]. This leads to a high demand of drying energy in paper coating applications and therefore higher costs. Therefore, this study additionally focuses on the investigation of properties relevant for coating applications such as rheology and surface tension at higher solids content to assess the possibility of using these biopolymers in industrial coating applications in the future. These high solids contents were chosen so that the viscosity of the biopolymer materials at high shear rates (50,000 s^−1^) relevant for industrial coating applications are comparable to the synthetic reference material (EVOH).

## 2. Materials and Methods

### 2.1. Materials and Solution Preparation

Aqueous alginate and chitosan solutions were prepared following Kopacic et. al. [39] To prepare an aqueous alginate solution, powdery alginic acid sodium salt with a predefined viscosity of 15–25 mPa·s at 1 wt% in water at 25 °C (from Sigma-Aldrich, Saint Louis, MO, USA)) was used. The solution was prepared by heating deionized water to 75 °C and by subsequently adding the desired alginic acid sodium salt amount to achieve a concentration of 5 wt%. To prevent agglomerations, alginate was added in small portions and stirred continuously. The solution was kept at the defined temperature for 6 h. The storage temperature of the aqueous alginate solution was 5 °C.

Chitosan powder (88–89% degree of deacetylation, BioLog Heppe, Landsberg, Germany) was used to prepare the solution. According to the specifications, a 1 wt% solution dissolved in a 1 wt% acetic acid has a dynamic viscosity lower than 30 mPa s. The powder was added to deionized water at 70 °C in small portions to again achieve a concentration of 5 wt%. After the solution was stirred for 6 h at 70 °C, the pH value was adjusted to four by adding acetic acid (Carl Roth, Karlsruhe, Germany, ≥99%) and stirred for another 4 h. The aqueous chitosan solution was then stored at 5 °C in the refrigerator.

Microfibrillated cellulose (MFC) produced from softwood pulp was purchased from Sappi Biotech (Gratkorn, Austria) at a solids content of 3.3 wt% and stored at 5 °C.

Granular ethylene vinyl alcohol (EVOH) was used to prepare the solution following the instructions provided by the manufacturer. The Exceval ethylene vinyl alcohol (from Kuraray, Hattersheim am Main, Germany) has 98–99 mol% of hydrolysis, and according to specifications, a 4 wt% aqueous solution has a viscosity of 3.5–4.5 mPa s at 20 °C. To prepare a 10 wt% solution, EVOH was added to deionized water at room temperature and continuously stirred until evenly dispersed. The mixture then was heated to 95 °C and kept at this temperature for 1–2 h. After preparation, the EVOH solution was slowly cooled down and stored at 50 °C until use.

### 2.2. Solution Properties

A halogen moisture analyzer HR73 (Mettler Toledo, Columbus, OH, USA) was used to measure the solids content. Solution densities were measured with a density meter DMA 4500M (Anton Paar, Graz, Austria). The pH values were determined using an inoLab pH 7110 (WTW, Weilheim, Germany). A data physics OCA200 (DataPhysics Instruments, Filderstadt, Germany) device was used to determine surface tension by the pendant drop method using a cannula diameter of 1.83 mm. For MFC, this method led to a separation of fibrils and water because the fibrous material is not pressed out at the same rate as the liquid phase, and thus, the surface tension of this material could not be evaluated. All solution properties were measured at ambient conditions of 23 °C and 50% RH. Viscosities were determined using a Physica MCR 301 rheometer (Anton Paar, Graz, Austria). Alginate, Chitosan and EVOH were measured at shear rates up to 50,000 s^−1^ in a cylindrical measuring system with a gap width of 0.1 mm and a gap length of 15 mm. MFC could not be measured in the cylindrical system and therefore was measured between two parallel plates with 50 mm diameter and a gap width of 1 mm at shear rates up to 1000 s^−1^.

### 2.3. Film Casting

To cast freestanding films, all mixtures were diluted to 1 wt% with deionized water. No plasticizers or other additives were added to the mixtures. The desired amount was poured into polystyrene Petri dishes to obtain films with a grammage of 40 ± 5 g/m^2^. The films were dried at room temperature for 72 h and afterwards conditioned in accordance to ISO 187 [40] (23 °C and 50% relative humidity) for at least 48 h prior to characterization.

### 2.4. Films Characterisation

#### 2.4.1. Optical Properties

Optical properties of the films were measured using a Datacolor Elrepho^®^ spectrophotometer (Lorentzen & Wettre, München, Germany). The transparent polymer films were measured on top of a white brightness standard, which was measured without a film placed on its surface for comparability. The L*, a* and b* values (CIE 1976 system) were determined according to ISO 5631-2:2022 [41].

#### 2.4.2. Thickness and Apparent Density

The basis weight of the films was measured using an analytical balance and the thickness was measured according to EN ISO 534 [42] at three different positions on each film. These measurements were performed on twenty films. To obtain the grammage of each individual film, the individual film weight was divided by the area of the Petri dishes. The apparent density was determined according to EN ISO 534 [42].

#### 2.4.3. Surface Properties

Several surface properties of the films were analyzed. The roughness was measured on ten individual films using a Parker Print Surf (PPS, Luhne Messtechnik, Jüchen, Germany) instrument according to TAPPI standard T 555 [43]. Surface wettability was investigated via contact angle measurements with deionized water using a Fibro Dat 1100 (Fibro System, Stockholm, Sweden) according to TAPPI standard T 558 [44]. For each type of film, 15 drops of 4 µL were measured.

#### 2.4.4. Mechanical Properties

The mechanical properties of the films were investigated using a universal tensile tester Zwick Z010 (ZwickRoell, Ulm, Germany). To prepare the samples for mechanical testing, ten strips of 15 mm width and at least 80 mm length were cut from the center of the round films. The tensile tests were performed according to EN ISO 1924-2 [45] with an initial clamp separation of 50 mm and at a test speed of 20 mm/min.

#### 2.4.5. Barrier Properties

Air permeability (Bendtsen) was examined on five different films according to DIN 53120-1. Water vapor transmission rate (WVTR) was determined on three films according to TAPPI standard T 448 [46] and oxygen transmission rate (OTR) on five films according to EN ISO 15105-2 [47] using a Perme OX2/230 oxygen transmission rate test system (Labthink, Jinan, China) at 50% RH and 23 °C. For each of the five films, the OTR measurement was repeated three times resulting in 15 measurements. Grease resistance was investigated by performing a KIT test on three films according to TAPPI standard T 559 cm^−12^ [48].

For all data, an outlier test was performed to remove values more than three scaled median absolute deviations (MAD) from the median. Mean values, 95% confidence intervals and number of outliers of all measured film properties are presented in Table A2, Table A3, Table A4 and Table A5 in the Appendix A.

## 3. Results

### 3.1. Biopolymer Solution Properties

**Figure 1:**
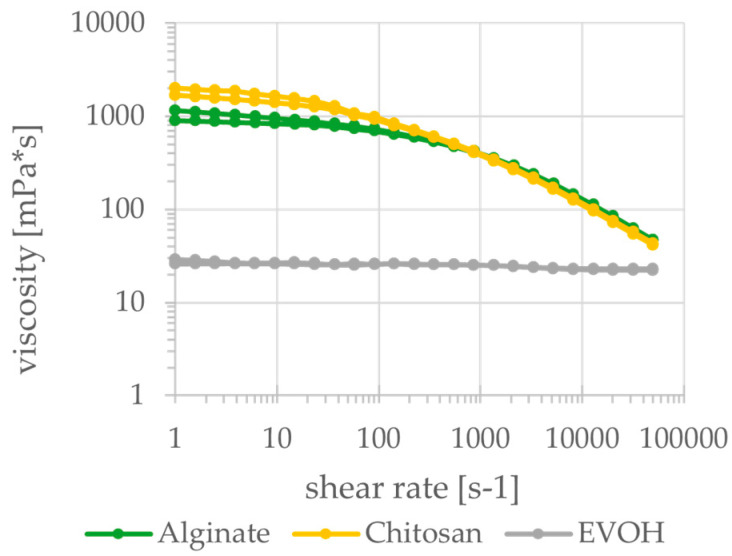
Viscosity of alginate, chitosan and EVOH dependent on shear rate (cylindrical measurement system).

**Figure 2:**
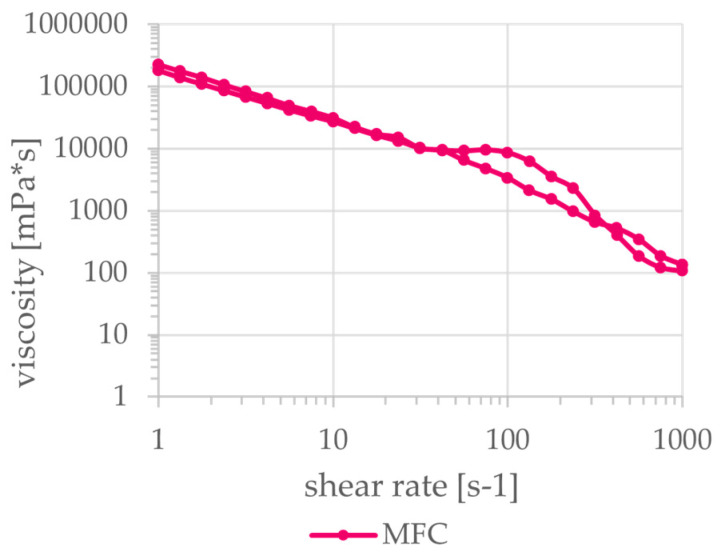
Viscosity of MFC dependent on shear rate (parallel plates measurement system).

Despite preparing the biopolymer solutions at higher solids content than comparable studies, solids contents of the biobased material mixtures shown in Table 1. are still considerably lower than the synthetic reference material ethylene vinyl alcohol (EVOH). For future applications in paper coatings, this would results in a higher total wet application amount for the same application weight of dry coating and therefore higher drying energy demand. At the chosen concentrations, the surface tension of alginate is slightly lower than of EVOH whilst the surface tension of chitosan is higher. Depending on potential application techniques (e.g., curtain or spray coating), this has to be considered. An important property in every coating process is the viscosity of the material at relevant shear rates. The viscosities of the materials are shown in Figure 1 and Figure 2. All three biobased materials have higher viscosities than the reference material especially at lower shear rates but also show distinct shear-thinning behavior. The reference material EVOH shows a less pronounced shear-thinning behavior, which, however, is difficult to detect given the scale of the y-axis in Figure 1. Thus, at higher shear rates, the biobased materials have similar viscosities as the reference material. For a successful and uniform application of these materials, a suitable application method preferably at high shear rates has to be chosen to reduce the viscosity accordingly. In general, the low solids content, the high viscosity and the pronounced shear-thinning behavior make the application of these biobased materials using established coating methods challenging.

### 3.2. Optical Properties

**Figure 3:**
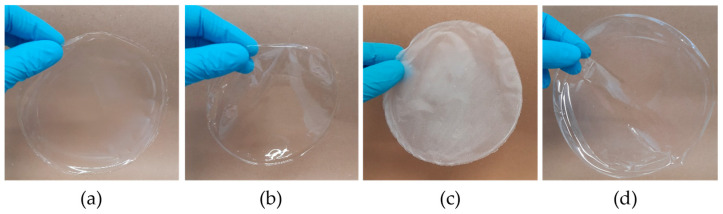
Alginate (**a**), Chitosan (**b**), MFC (**c**) and EVOH (**d**) solution cast films.

**Figure 4:**
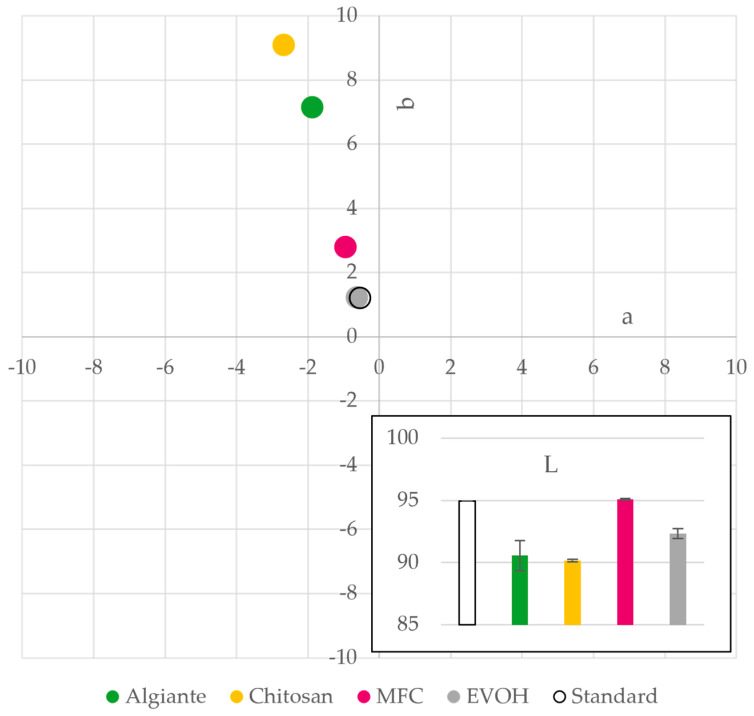
Optical properties (L*, a*, b*-values) of alginate, chitosan, MFC and EVOH solution cast films including measurement of a standard (n = 3).

Depending on the application, optical appearance of films is of interest. Alginate (a), chitosan (b) and EVOH (d) form transparent films as shown in Figure 3. Alginate and chitosan films, while transparent, have a yellow–green tint that is not detectable visually in the images depicted in Figure 3, but can be clearly seen in Figure 4, with high b* and higher negative a* value in the Elrepho measurements. Both alginate and chitosan films are also less bright (lower lightness L*) than the also transparent EVOH film. These apparent optical properties might limit the application possibilities of alginate and chitosan as pure films, but should not hinder application as a coating material on paper. Contrary to the transparent appearance of the other three materials, microfibrillated cellulose (MFC) films (c) have a significantly higher opacity and will thus strongly disturb the visibility of packaged goods, when pure films are used. For paper coating application, this higher opacity is no hindrance, as paper is opaque anyway. Since it is bleached MFC, the brightness is higher than for the other films. It can be concluded that alginate and MFC may not be suitable for all applications due to their optical appearance if pure films are used and undisturbed visibility of products is desired, but for paper coating application, all materials are suitable.

### 3.3. Thickness and Density

**Figure 5:**
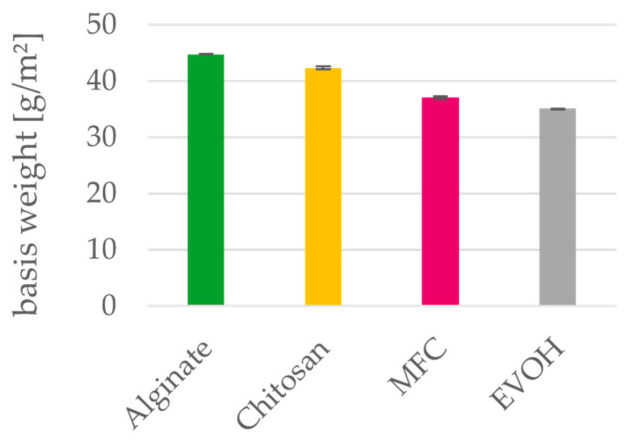
Basis weight of solution cast alginate, chitosan, MFC and EVOH films. Mean value and 95% confidence interval (n = 20).

**Figure 6:**
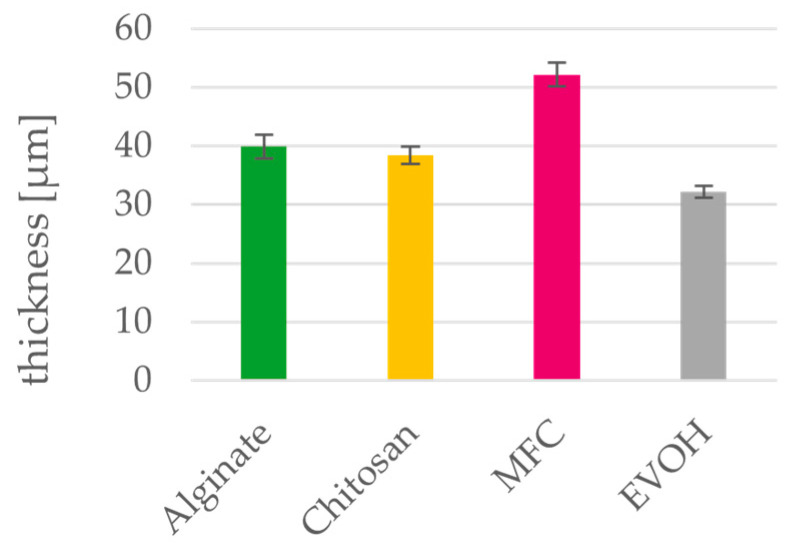
Thickness of solution cast alginate, chitosan, MFC and EVOH films. Mean value and 95% confidence interval (n = 20).

**Figure 7:**
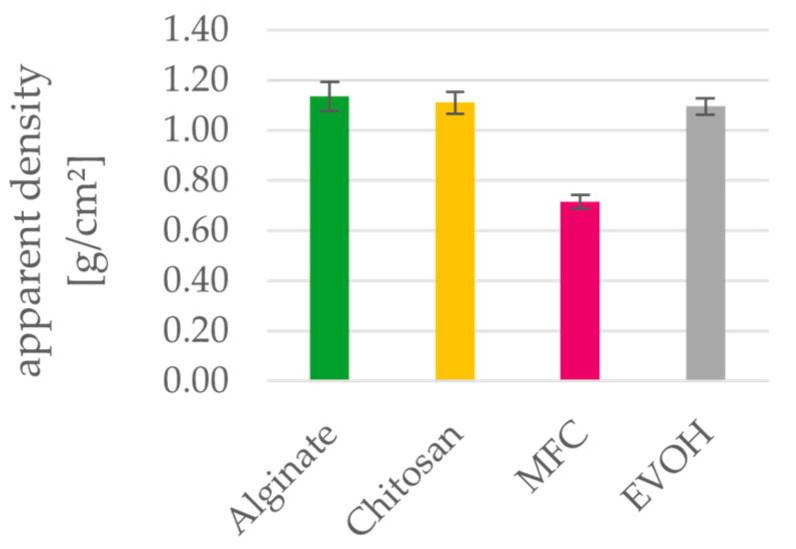
Apparent density of solution cast alginate, chitosan, MFC and EVOH films. Mean value and 95% confidence interval (n = 20).

Figure 5, Figure 6 and Figure 7 depict basis weight, thickness and apparent densities of the films. Regarding their calculated apparent densities, both alginate and chitosan can be compared to the petrochemical reference material EVOH. MFC films have a higher thickness and therefore have a lower apparent density of 0.72 compared to the other materials. Density values of MFC films in the literature [24,49] range between 0.8 g/cm³ and 1.1 g/cm³ [23,50]. This discrepancy is caused by the high measured thickness of MFC films, which can be ascribed to the wrinkling of the films during the drying process. These investigation of the materials leads to the conclusion that the required thickness of the biopolymer coating layers to be applied on paper will not differ from those of the established EVOH.

### 3.4. Surface Properties

**Figure 8:**
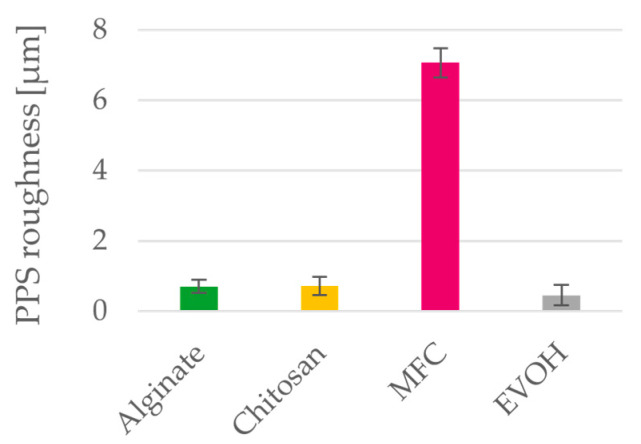
Roughness of solution cast alginate, chitosan, MFC and EVOH films. Mean value and 95% confidence interval (n = 10).

Except for MFC, all materials form very smooth films as is demonstrated by the PPS roughness depicted in Figure 8. MFC films have a high PPS roughness of 7 µm while all other films exhibit values below 1 µm. Reasons for this high PPS roughness is significant shrinkage of the MFC films during free drying of the films and to some extent also the particulate nature of the MFC fibrils.

**Figure 9:**
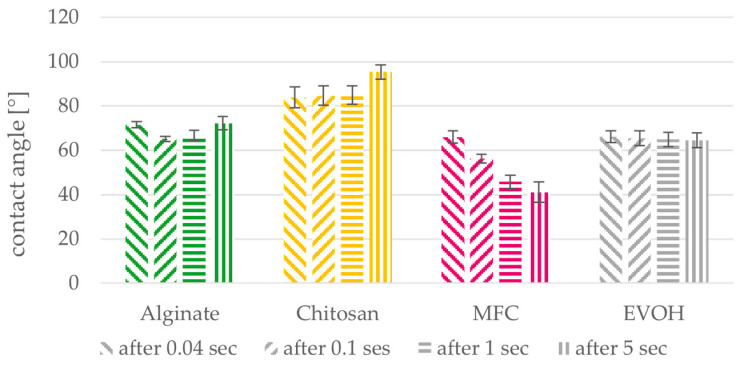
Water contact angles on alginate, chitosan, MFC and EVOH solution cast films after 0.04, 0.1, 1 and 5 s. Mean values and 95% confidence intervals (n = 15).

Figure 9 shows the behavior of water drops applied on the films after different times. Both alginate and chitosan show little time-dependent behavior at the beginning but start to show an increase in contact angle after the first five seconds. On MFC films, due to the hydrophilic nature of cellulose, contact angles decrease significantly within the first seconds. For EVOH films, no distinct change in contact angle is noticed in the first seconds after drop application.

**Figure 10:**
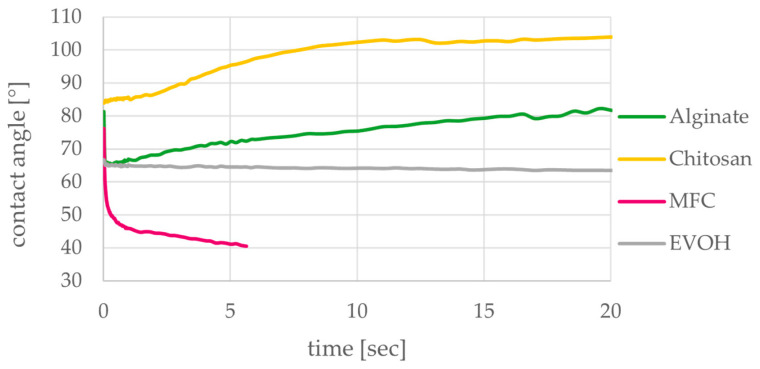
Progression of water contact angles on alginate, chitosan, MFC and EVOH solution cast films for the duration of 20 s showing swelling of alginate and chitosan films (n = 15).

Figure 10 shows the contact angles for drops of deionized water on the films for the duration of 20 s. All contact angles start below 90° indicating wetting of the surface and hydrophilicity. While contact angles on EVOH films stay constant after the initial drop application, the other materials show distinct time-dependent behavior. The decrease in contact angle on MFC films was so rapid that measured drops could no longer be detected after approximately 6 s because the contact angle became too low. At very low contact angles, the drops can no longer be measured because the software can no longer detect the correct contour line of the drop. This is in part due to the surface roughness of the films, which makes drop detection more difficult. While fast wetting of the surface was observed due to the hydrophilic nature of MFC films, we observed that penetration into the MFC films was only noticed to some extent because a lack of porosity hinders the water from entering into the film.

**Figure A1:**
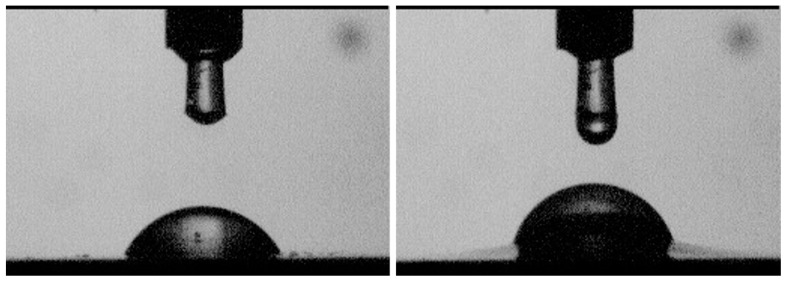
Water contact angle measurement on alginate solution cast film after 0.01 s (**left**) and 20 s (**right**). Right image shows the swelling induced rise of the surface on which the drop lies leading to higher detected contact angles as the baseline of the measurement stays the same.

On alginate and chitosan films, the contact angle shows an increase after the initial wetting of the surface. This behavior results from the swelling and partial dissolution of the films, which falsifies the detection of the drop. The swelling causes the surface on which the drop lies to rise, while the baseline of the measurement stays the same, leading to higher detected contact angles (see also Figure A1). On alginate films, the drop can even create a hole in the film when too much of the film is dissolved. For this reason, two drops in addition to the outliers had to be excluded from the analysis. Chitosan films show more swelling than alginate films within the first 10 s. But the films still prevent all applied drops from penetrating through the material during the measurement time of 20 s. According to the initial contact angle after 0.1 s shown in Figure 9 And before the start of swelling, alginate and chitosan films are less hydrophilic than the EVOH films and especially MFC films. Due to their hydrophilic behavior when in contact with water, alginate, chitosan and MFC on their own are not applicable as packaging materials for moist products and only of limited applicability for packaging of food in general. Regarding coating of paper, additional components would have to be added to these materials or a multilayer coating would have to be applied to fulfil the desired requirements. On the other hand, the hydrophilicity of the evaluated materials can be considered as beneficial when recyclability of the materials coated on paper is considered.

### 3.5. Mechanical Properties

**Figure 11:**
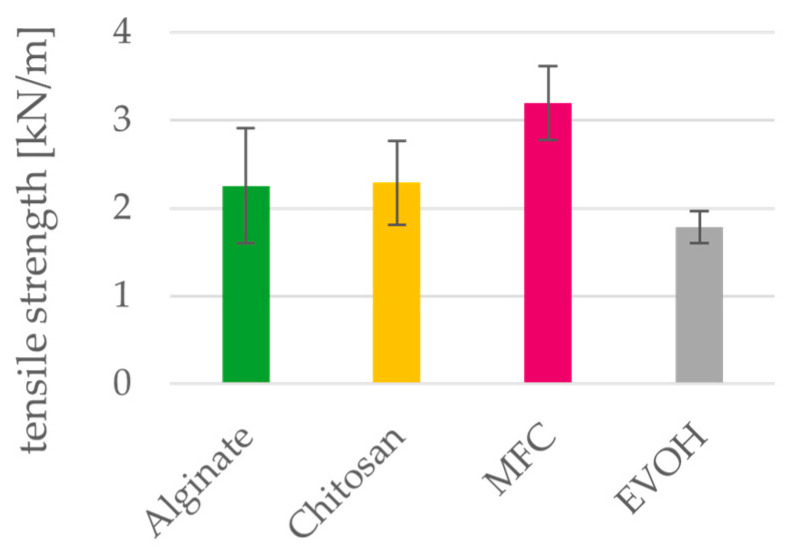
Tensile strength of alginate, chitosan, MFC and EVOH solution cast films. Mean values and 95% confidence intervals (n = 10).

**Figure 12:**
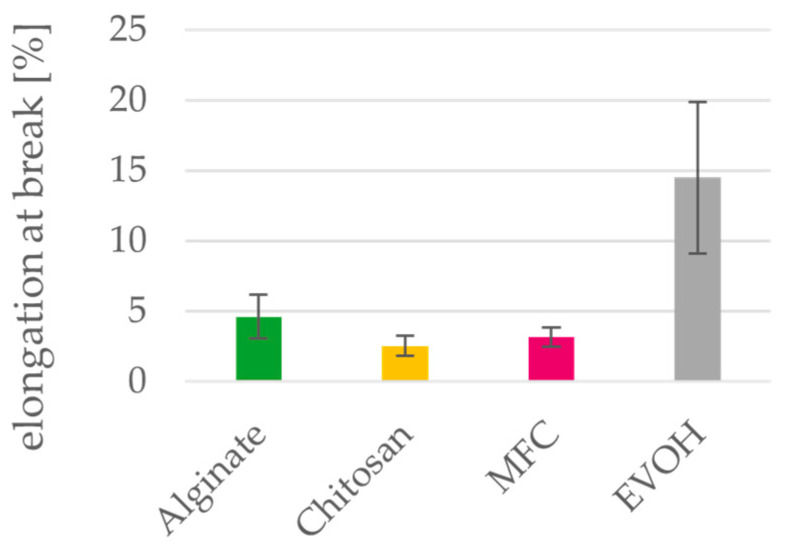
Elongation at break of alginate, chitosan, MFC and EVOH solution cast films. Mean values and 95% confidence intervals (n = 10).

The mechanical properties of the cast films are shown in Figure 11 and Figure 12. Tensile strength of MFC films is significantly higher than for EVOH films, and while alginate and chitosan also have higher tensile strength, the difference cannot be considered as significant. The elongation at break is significantly lower for all three biopolymers compared to the petrochemical reference material EVOH, indicating a more brittle behavior. For this reason, in most studies, they are mixed with plasticizers to improve their flexibility and handling, which in turn can reduce their tensile strength [5]. Regarding their application as a coating material for paper, there is a need to modify the barrier-coating formulation to improve the elongation at break but at the same time keep the tensile strength high to achieve good processability.

### 3.6. Barrier Properties

All films have air permeabilities (Bendtsen) of 0 mL/min and a grease resistance value of 12 according to the KIT test (see Table A1 in the Appendix A). Thus, all tested materials are good air and grease barriers and show the potential to be used as packaging for greasy goods.

**Figure 13:**
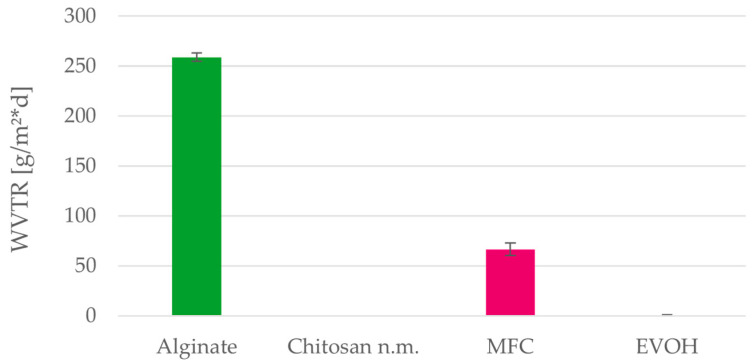
Water vapor transmission rate (WVTR) of alginate, MFC and EVOH solution cast films. Mean values and 95% confidence intervals (n = 3).

The water vapor transmission rate (WVTR) could not be measured for the chitosan films as they could not withstand the temperatures caused by the hot wax seal required by this method. The resulting cracks in the film prevented the measurement. Performed tests show that alginate has the highest and EVOH the lowest WVTR of the considered materials (Figure 13). The WVTR of alginate is high compared to the reference material EVOH but can still provide a sufficient barrier for some packaging applications. MFC films have transmission rates below 70 g/m²·d and can therefore be used for packing foodstuff more sensitive to water vapor.

**Figure 14:**
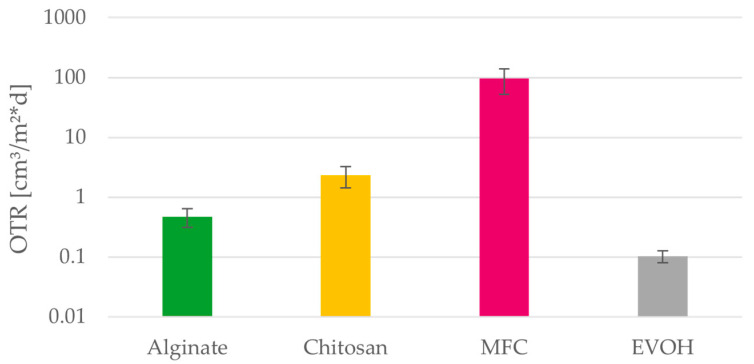
Oxygen transmission rate (OTR) of alginate, chitosan, MFC and EVOH solution cast films on a logarithmic scale. Mean values and 95% confidence intervals (n = 15).

The measured oxygen transmission rates (OTR) shown in Figure 14 depict the mean values and confidence intervals of three measurements on five individual films. Because the deviations between individual films are very high, the detection and exclusion of outliers is very important. One film produced from EVOH has an OTR of 47 cm³/m²·d although this material is known for its excellent oxygen barrier properties, and the remaining films have OTR values below 0.15 cm³/m²·d. The variance between the individual measurements cannot be linked to any of the other measured parameters like air permeability or thickness of the individual films and is most likely caused by pinholes in the µm range. These can be caused by the films preparation or handling and have been shown to decrease the oxygen barrier of polymer films [49]. The oxygen transmission rates of alginate and chitosan are not as low as the results for EVOH films but sufficient even for food with high requirements regarding oxygen transmission. This leads to the conclusion that with the right application and handling the materials provide excellent oxygen barriers and are suitable for coating packaging materials of oxygen-sensitive food.

With regard to the application of these materials as a barrier coating for paper and board, it should be considered that the thickness of the layers applied on paper and board is significantly lower than the thickness of the pure films, which of course will also tend to reduce the barrier properties.

## 4. Conclusions

Whilst many studies focus on the optimization of barrier or tensile properties of a certain biopolymer film or the combination of biopolymers with other materials, this research focuses on the properties of individual pure biopolymers in order to evaluate their applicability in the barrier coating of paper and board. In this research, alginate, chitosan and MFC solution cast films were investigated for barrier applications on paper and compared to EVOH as a synthetic reference material.

All surveyed biopolymers exhibit brittle material behavior with tensile strength higher or as high as EVOH and lower elongation at break than the reference material. Therefore, applications requiring specific mechanical properties regarding processability will need additional components like plasticizers. Contact angles of alginate and chitosan increase over time because of swelling of these materials. Alginate has higher WVTR than MFC and EVOH and both alginate and chitosan have low OTR. These materials applied on paper or board as a barrier layer therefore show the potential to be used in a multitude of packaging applications where oxygen transmission is the main requirement and water vapor transmission is not crucial or to be a part of multilayer coating. The contact angle of MFC films is steadily decreasing, and therefore, this material is not suited for moist products, but because of its low WVTR, it may be adequate as a water vapor barrier in multilayer coating. OTR of MFC is not as low as that of other tested biopolymers but is still on a level that is suitable for a wide range of packaging applications.

Based on these identified properties, none of the surveyed materials in their pure form can be used as a packaging film, but they all provide promising properties for future applications in the packaging industry as barrier coating materials for paper and board.

## Figures and Tables

**Table 1:** Properties (solids content, density, pH value, surface tension) of alginate, chitosan, microfibrillated cellulose (MFC) and ethylene vinyl alcohol (EVOH) (n.m., not measurable).

Material	Solids Content (wt%)	Density (g/cm³)	pH	Surface Tension (mN/m)
Alginate	5.10	1.025	6.95	53.47
Chitosan	4.92	1.019	4.85	67.87
MFC	3.30	1.001	7.23	n.m.
EVOH	10.30	1.008	5.10	56.769

## Data Availability

Not applicable.

## Appendix A

**Table A1:** Optical properties (L*, a*, b*-values) of alginate, chitosan, MFC and EVOH solution cast films including measurement of a standard.

Material	L*	a*	b*
Standard	94.95 ± 0	−0.53 ± 0.03	1.2 ± 0.02
Alginate	90.57 ± 1.21	−1.87 ± 0.69	7.13 ± 3.52
Chitosan	90.17 ±0.09	−2.66 ± 0.11	9.08 ± 0.35
MFC	59.09 ± 0.05	−0.93 ± 0,14	2.78 ± 0.41
EVOH	92.34 ± 0.41	−0.62 ± 0.03	1.12 ± 0.08

**Table A2:** Basis weight (n = 20), thickness (n = 20), apparent densities (n = 20) and roughness (n = 10) of solution cast films. Mean value ± 95% confidence interval.

Material	Basis Weight (g/m²)	Thickness (µm)	Apparent Density (g/cm³)	PPS Roughness (µm)
Alginate	44.80 ± 0.04 (one outlier)	39.90 ± 2.05	1.14 ± 0.06	0.71 ± 0.19
Chitosan	42.33 ± 0.24	38.37 ± 1.47	1.11 ± 0.04	0.72 ± 0.26
MFC	37.07 ± 0.25	52.17 ± 2.07	0.72 ± 0.03	7.06 ± 0.42
EVOH	35.10 ± 0.03	32.16 ± 0.96 (one outlier)	1.10 ± 0.03 (one outlier)	0.45 ± 0.29

**Table A3:** Water contact angles of solution cast films after 0.04 s, 0.1 s, 1 s and 5 s. Mean values and 95% confidence intervals (n = 15).

Material	Contact Angle after 0.04 s (°)	Contact Angle after 0.1 s (°)	Contact Angle after 1 s (°)	Contact Angle after 5 s (°)
Alginate (three outliers)	71.55 ± 1.38	65.16 ± 1.22	66.69 ± 2.29	72.24 ± 3.09
Chitosan	83.93 ± 4.71	84.69 ± 4.47	84.88 ± 4.13	95.37 ± 3.25
MFC (two outliers)	65.97 ± 2.81	56.21 ± 2.00	45.80 ± 2.92	41.08 ± 4.65
EVOH	66.17 ± 2.70	65.46 ± 3.37	64.92 ± 3.17	64.52 ± 3.34

**Table A4:** Tensile strength (n = 10), elongation at break (n = 10), water vapor transition rate (n = 3) and oxygen transmission rate (n = 15) of solution cast films. Mean values and 95% confidence intervals.

Material	Tensile Strength (kN/m)	Elongation at Break (%)	WVTR (g/m²·d)	OTR (cm³/m²·d)
Alginate	2.25 ± 0.66	4.60 ± 1.56	258.95 ± 4.24 (one outlier)	0.47 ± 0.16 (six outliers)
Chitosan	2.29 ± 0.48 (one outlier)	2.53 ± 0.70 (three outliers)	-	2.32 ± 0.89 (three outliers)
MFC	3.20 ± 0.42	3.19 ± 0.67	66.45 ± 6.25	96.40 ± 43.62 (three outliers)
EVOH	1.78 ± 0.18	14.51 ± 5.40	0.45 ± 0.40 (one outlier)	0.10 ± 0.02 (three outliers)

**Table A5:** Air permeability (n = 10) and grease resistance (n = 3) of solution cast films.

Material	Air Permeability (mL/min)	Grease Resistance KIT
Alginate	0	12
Chitosan	0	12
MFC	0	12
EVOH	0	12
